# A novel approach to treating post-stroke depression: administration of Botulinum Toxin A via local facial injection

**DOI:** 10.3389/fneur.2024.1372547

**Published:** 2024-06-18

**Authors:** Xiao-Yan Feng, Ting-Ting Shen, Qian-Chang Wu, Jun Wang, Ping Ni, Jing Liu, Xu-Ping Zhou, Hua Hu, Wei-Feng Luo

**Affiliations:** ^1^Department of Neurology and Clinical Research Center of Neurological Disease, The Second Affiliated Hospital of Soochow University, Suzhou, China; ^2^Department of Neurology, Wuxi No.2 People's Hospital, Jiangnan University Medical Center, Wuxi, China

**Keywords:** post-stroke depression, Sertraline, Botulinum Toxin A, local facial injection, treatment

## Abstract

**Background:**

Post-stroke depression (PSD) is a frequent complication following a stroke, characterized by prolonged feelings of sadness and loss of interest, which can significantly impede stroke rehabilitation, increase disability, and raise mortality rates. Traditional antidepressants often have significant side effects and poor patient adherence, necessitating the exploration of more suitable treatments for PSD. Previous researchers and our research team have discovered that Botulinum Toxin A (BoNT-A) exhibits antidepressant effects. Therefore, our objective was to assess the efficacy and side effects of BoNT-A treatment in patients with PSD.

**Methods:**

A total of 71 stroke patients meeting the inclusion criteria were allocated to the two group. 2 cases were excluded due to severe neurological dysfunction that prevented cooperation and 4 cases were lost follow-up. Ultimately, number of participants in the BoNT-A group (*n* = 32) and Sertraline group (*n* = 33). Treatment efficacy was evaluated 1, 2, 4, 8 and 12 weeks post-treatment.

**Results:**

There were no significant differences in baseline characteristics between the two groups (*p* > 0.05). Both groups exhibited comparable treatment efficacy, with fewer side effects observed in the BoNT-A group compared to the Sertraline group. BoNT-A therapy demonstrated significant effects as early as the first week (*p* < 0.05), and by the 12th week, there was a notable decrease in neuropsychological scores, significantly lower than the baseline level. The analysis revealed significant differences in measurements of the Hamilton Depression Scale (HAMD) (*F*(770) = 12.547, *p* = 0.000), Hamilton Anxiety Scale (HAMA) (*F*(951) = 10.422, *p* = 0.000), Self-Rating Depression Scale (SDS) (*F*(1385) = 10.607, *p* = 0.000), and Self-Rating Anxiety Scale (SAS) (*F*(1482) = 11.491, *p* = 0.000).

**Conclusion:**

BoNT-A treatment effectively reduces depression symptoms in patients with PSD on a continuous basis.

## Introduction

Stroke is a cerebrovascular event resulting from a sudden interruption of blood supply to the brain, causing irreversible tissue damage. This condition encompasses thrombotic, embolic, or hemorrhagic events. A common complication of stroke is post-stroke depression (PSD), a mood disorder characterized by persistent emotional depression and loss of interest (1). Psychiatrists have observed PSD for nearly a century, and since the 1970s, numerous studies have been conducted to investigate this condition. PSD is a prevalent and manageable complication of stroke (2), with meta-analyses estimating its prevalence to range from 18 to 33% (3, 4). Werheid discovered an intriguing pattern where depressive symptoms in stroke patients tend to worsen in the first 6 months, improve within a year, and then worsen again after the second year (5). PSD can increase disability and mortality rates, reduce rehabilitation efficiency, and lead to a decline in motor function and quality of life, placing a burden on both families and society (6). The clinical manifestations of PSD can be classified into core and non-core symptoms. Core symptoms consist of low mood, anhedonia, and fatigue, while non-core symptoms include cognitive impairment, sleep disturbances, unexplained pain, sexual dysfunction, appetite changes, and gastrointestinal issues. However, due to the diverse and non-specific nature of these symptoms, diagnosis and treatment of PSD are often overlooked or delayed (2).

Research studies have shown that prompt administration of antidepressant therapy after a stroke can prevent the development of PSD (7). Additionally, antidepressant therapy can improve the prognosis of stroke patients, including cognitive and executive functions, thereby enhancing their quality of life (8). It is worth noting that a randomized placebo-controlled trial has established that the benefits of antidepressants can extend beyond emotional symptoms. Patients who received antidepressants demonstrated better motor recovery compared to the control group, leading to a significant increase in the proportion of patients achieving partial or complete living independence (9). As a result, early detection of PSD and timely use of antidepressants are crucial for effective stroke management. However, traditional antidepressants often fail to meet clinical needs due to their slow onset of action and adverse side effects, such as hepatotoxicity, nephrotoxicity, gastrointestinal discomfort, cognitive decline, etc. (10). An increasing number of studies have demonstrated the efficacy of Botulinum Toxin A (BoNT-A) in the treatment of depression. Clinical randomized controlled trials have consistently confirmed the safety and effectiveness of BoNT-A as an antidepressant therapy (11). Building upon this previous research, we recruited PSD patients who met the inclusion criteria to receive BoNT-A for antidepressant treatment, with the traditional antidepressant Sertraline serving as the control group in our study.

## Materials and methods

### Study population and setting

**Figure 1:**
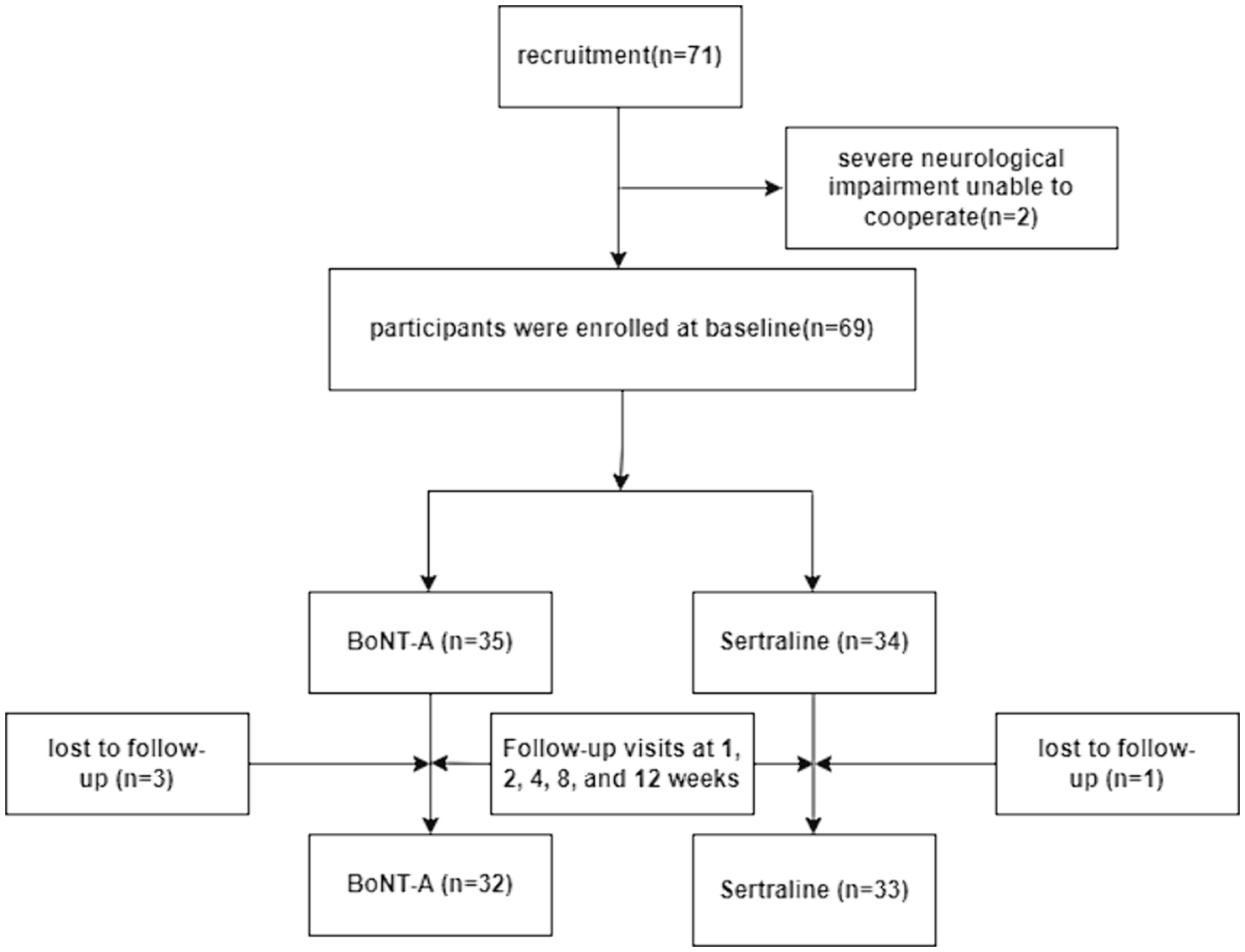
Patient recruitment, grouping, and assessment treatment flowchart.

A total of 71 patients with PSD were enrolled in this study, conducted from February 2020 to January 2023. The patients were recruited from the Department of Neurology at the Second Affiliated Hospital of Suzhou University and the Fifth People’s Hospital of Zhangjiagang City. The patient recruitment, grouping, and treatment assessment flowchart is shown in Figure 1.

The inclusion criteria were as follows: (1) patients with confirmed stroke based on head CT or head MRI; (2) patients who met the diagnostic criteria outlined in the fifth edition of the Diagnostic and Statistical Manual of Mental Disorders (DSM-5); (3) patients with mild to moderate PSD, aged between 18 and 80 years; (4) patients who experienced stroke within 2–6 months prior to the study and exhibited depressive symptoms lasting for more than 2 weeks; (5) patients with a score ranging from 7 to 24 on the 17-item Hamilton Depression Scale (HAMD-17); (6) patients who, along with their family, provided signed consent forms.

The exclusion criteria for this study were as follows: (1) individuals with a HAMD-17 score higher than 24 points; (2) individuals with neurological and psychiatric disorders not associated with PSD, and those who had previously used antidepressants; (3) individuals with aphasia, comprehension disorders, or cognitive dysfunction that would impede test completion; (4) individuals with a history of brain injury, epilepsy, or Parkinson’s disease; (5) individuals with significant systemic diseases, such as immune system disorders; (6) individuals with severe liver or kidney dysfunction.

We performed a 1:1 simple randomization of all enrolled patients to an intervention group with BoNT-A injection and to a control group with Sertraline medication. Specific research workers were set for subjects recruitment, randomizing and pharmacological intervention. Professional clinicians were responsible for psychological scale assessments which were masked to assignment scheme.

### Pharmacological intervention

**Figure 2:**
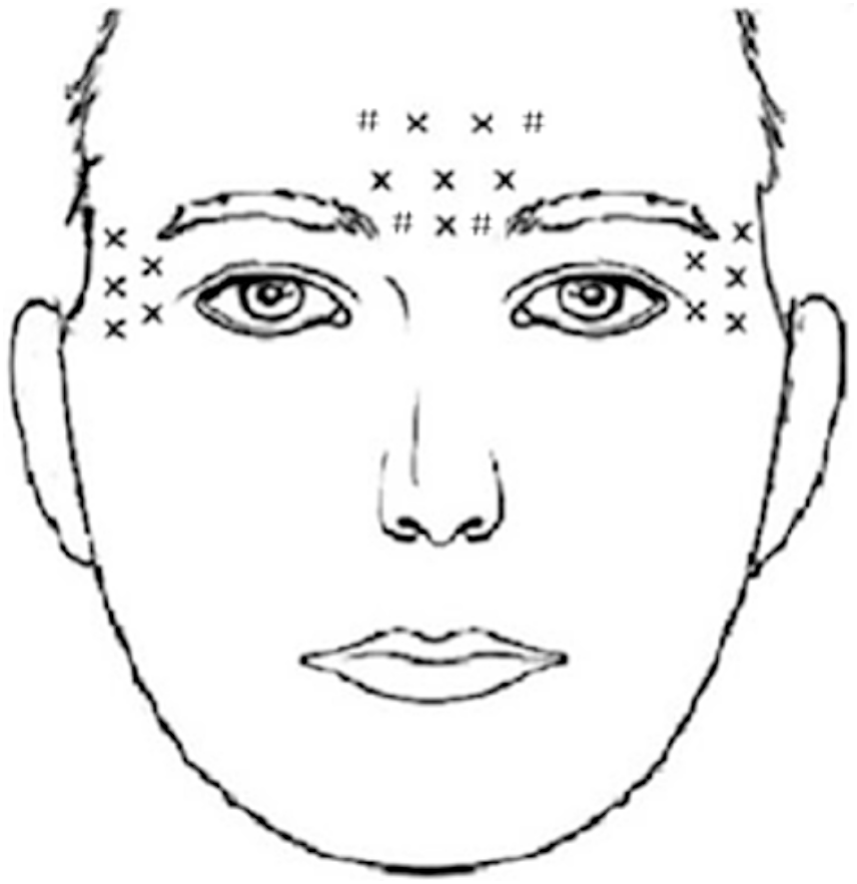
A schematic diagram of BoNT-A injection sites. The X marks the original injection sites, while the # marks the additional ones we have identified. The occipital frontalis muscle, frowning muscle, and depressor muscle each received 10 injections, and the bilateral lateral canthus and temporal regions each received 5 injections. In total, 20 sites were injected with BoNT-A, with a dosage of 5 units per site, resulting in a cumulative dose of 100 units.

The BoNT-A injection group received locally injected BoNT-A (Lanzhou, China, trade name: Hengli; trade number: S1097003). The total injection unit was 100 units, diluted to 2 mL of physiological saline at a concentration of 0.9%. Local injections were administered to 20 areas on the forehead and bilateral temporal regions, with a dosage of five units per area. 10 areas were injected into these muscles such as the procerus, corrugator, and frontalis (frontal part of the occipitofrontalis muscles) muscles, 10 areas were injected into lateral canthus of the eyes and the bilateral temporal region (Figure 2). In the control group, patients were orally administered Sertraline, a first-line antidepressant drug produced in the United States, under the trade name “Zoloft” with the trade number H1048017. The dosage of Sertraline was 50–100 mg once daily in the morning for 12 weeks.

### Treatment assessments and analysis

To evaluate the treatment effect, various psychological scales were employed, including the HAMD-17, the Self-Rating Depression Scale (SDS), the 14-item Hamilton Anxiety Scale (HAMA-14), and the Self-Rating Anxiety Scale (SAS). The patients underwent psychological scale assessments at the baseline, 1st, 2nd, 4th, 8th, and 12th weeks. All assessing doctors received uniform, rigorous and standardized training on the scales to ensure the accuracy and consistency of patient scores.

Statistical analysis was performed using SPSS 19.0, with a significance level set at *p* < 0.05. Mean and standard deviation were used to describe the quantitative data at normal distribution. The baseline data between the two groups were compared using an unpaired *t*-test. For each group, the scores of HAMD, SDS, HAMA, and SAS were compared using One-way ANOVA followed by the Bonferroni *post hoc* correction. The scores between the BoNT-A group and the Sertraline group were compared using two-way repeated-measures ANOVA, followed by the Bonferroni *post hoc* correction.

We use the 12-week time point as the evaluation endpoint. During this period, the trial will be terminated if the patient develops any discomfort or requires other treatment options. Observe whether patients have allergic reactions within 0.5 h after the end of BoNT-A treatment, and evaluate the side effects of treatment in both groups after 12 weeks of treatment. Keep communication open with the patient during test periods.

## Results

### Baseline characteristics

A total of 71 stroke patients meeting the inclusion criteria were allocated to the two group. 2 cases were excluded due to severe neurological dysfunction that prevented cooperation and 4 cases were lost follow-up. Ultimately, 32 patients were enrolled in the BoNT-A, and 33 patients were enrolled in the Sertraline group. The baseline data, including age, sex, and scores of HAMD, SDS, HAMA, and SAS before treatment (Table 1), did not significantly differ between the two groups. The scoring data for all patients were followed up over 12 weeks.

**Table 1:** Comparison of baseline data between the two groups of PSD patients.

	BoNT-A (*n* = 32)	Sertraline (*n* = 33)	*p*	Unpaired *t*-test
Age (Year, mean ± SD)	67.53 ± 10.08	65.76 ± 9.69	0.472	0.723
Gender (female) [*n* (%)]	17 (53.10%)	24 (72.7%)	0.127^a^	
HAMD (Score, mean ± SD)	11.63 ± 3.26	11.30 ± 3.88	0.719	0.362
HAMA (Score, mean ± SD)	10.81 ± 3.65	11.36 ± 6.33	0.668	0.432
SDS (Score, mean ± SD)	39.94 ± 6.99	38.85 ± 6.52	0.518	0.650
SAS (Score, mean ± SD)	40.56 ± 6.56	39.42 ± 6.89	0.498	0.682

HAMD, Hamilton Depression Scale; SDS, Self-Rating Depression Scale; HAMA, Hamilton Anxiety Scale; SAS, Self-Rating Anxiety Scale; BoNT-A, Botulinum Toxin A.

a Fisher’s exact test.

### Efficiency of BoNT-A

**Figure 3:**
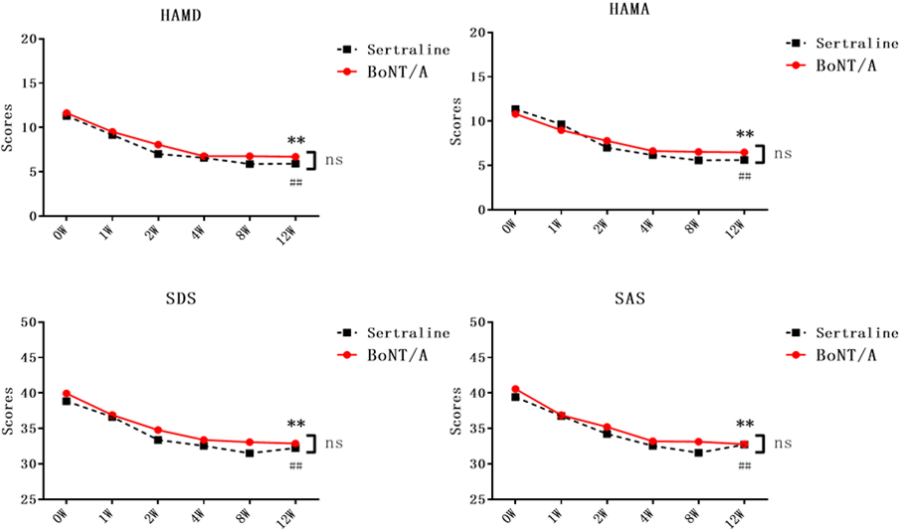
Comparison of neuropsychological scores between the two groups of PSD patients after treatment. The neuropsychological scores of both groups were subjected to two-way repeated-measures ANOVA. Results showed a significant difference between the BoNT-A group and the baseline after 12 weeks of treatment (**p < 0.01, *n* = 32), while the Sertraline group also exhibited a statistical difference from the baseline after 12 weeks of treatment (*##p <* 0.01, *n* = 33). Unpaired *t*-tests were conducted to compare the two groups at each time point, with non-significant results (ns, *p >* 0.05). HAMD, Hamilton Depression Scale; SDS, Self-Rating Depression Scale; HAMA, Hamilton Anxiety Scale; SAS, Self-Rating Anxiety Scale; BoNT-A, Botulinum Toxin A.

Following a 12-week treatment and follow-up period, the psychological scores of PSD patients in both the BoNT-A and Sertraline groups showed significant reductions compared with the baseline levels (*p* < 0.05). The HAMD score for the BoNT-A group decreased from 11.63 ± 3.26 (baseline) to 6.69 ± 3.50 (after 12 weeks of treatment), while the HAMD score for the Sertraline group decreased from 11.30 ± 3.88 (baseline) to 5.91 ± 3.21 (after 12 weeks of treatment). These findings indicate that the effectiveness of BoNT-A in treating depression is comparable to that of Sertraline, with no significant statistical difference observed between the two groups (Table 2; Figure 3).

**Table 2:** Comparison of the neuropsychological scores between the two groups of PSD patients at the baseline, 1st, 2nd, 4th, 8th, and 12th weeks.

	BoNT-A (*n* = 32)						Sertraline (*n* = 33)						*p*
	Baseline	1st week	2nd week	4th week	8th week	12th week	Baseline	1st week	2nd week	4th week	8th week	12th week	
HAMA (Score, mean ± SD)	10.81 ± 3.65	8.97 ± 3.93	7.78 ± 3.11	6.63 ± 2.92	6.53 ± 4.25	6.47 ± 3.63	11.36 ± 6.33	9.64 ± 5.73	7.00 ± 3.30	6.15 ± 2.94	5.58 ± 2.65	5.61 ± 3.18	0.736
HAMD (Score, mean ± SD)	11.63 ± 3.26	9.50 ± 3.37	8.06 ± 2.98	6.75 ± 2.97	6.75 ± 3.90	6.69 ± 3.50	11.30 ± 3.88	9.15 ± 3.81	7.00 ± 3.73	6.58 ± 3.14	5.88 ± 3.17	5.91 ± 3.21	0.971
SDS (Score, mean ± SD)	39.94 ± 6.99	36.91 ± 5.98	34.78 ± 4.94	33.38 ± 4.41	33.06 ± 6.50	32.88 ± 5.81	38.85 ± 6.52	36.64 ± 5.65	33.39 ± 4.87	32.55 ± 4.32	31.52 ± 3.95	32.24 ± 4.93	0.987
SAS (Score, mean ± SD)	40.56 ± 6.56	36.88 ± 6.74	35.22 ± 4.64	33.19 ± 4.39	33.13 ± 5.67	32.78 ± 5.00	39.42 ± 6.89	36.76 ± 5.29	34.24 ± 3.75	32.55 ± 3.76	31.58 ± 4.56	32.73 ± 5.51	0.963

In the BoNT-A treatment group, there were significant reductions in the scores of HAMD, SDS, HAMA, and SAS starting from the first week of treatment (*p* < 0.05). In the Sertraline group, the scores for HAMD and SAS showed a decrease from the first week of treatment, while the scores for HAMA and SDS decreased from the second week onwards (*p* < 0.05). Notably, the HAMA and SDS scores showed an earlier onset of improvement in comparison to the Sertraline group, however, there does not seem to be a difference in the change between the groups (Table 2; Figure 3). There was no significant statistical difference in the scores of all psychological scales between the two groups whenever assessed.

### Side effects

**Figure 4:**
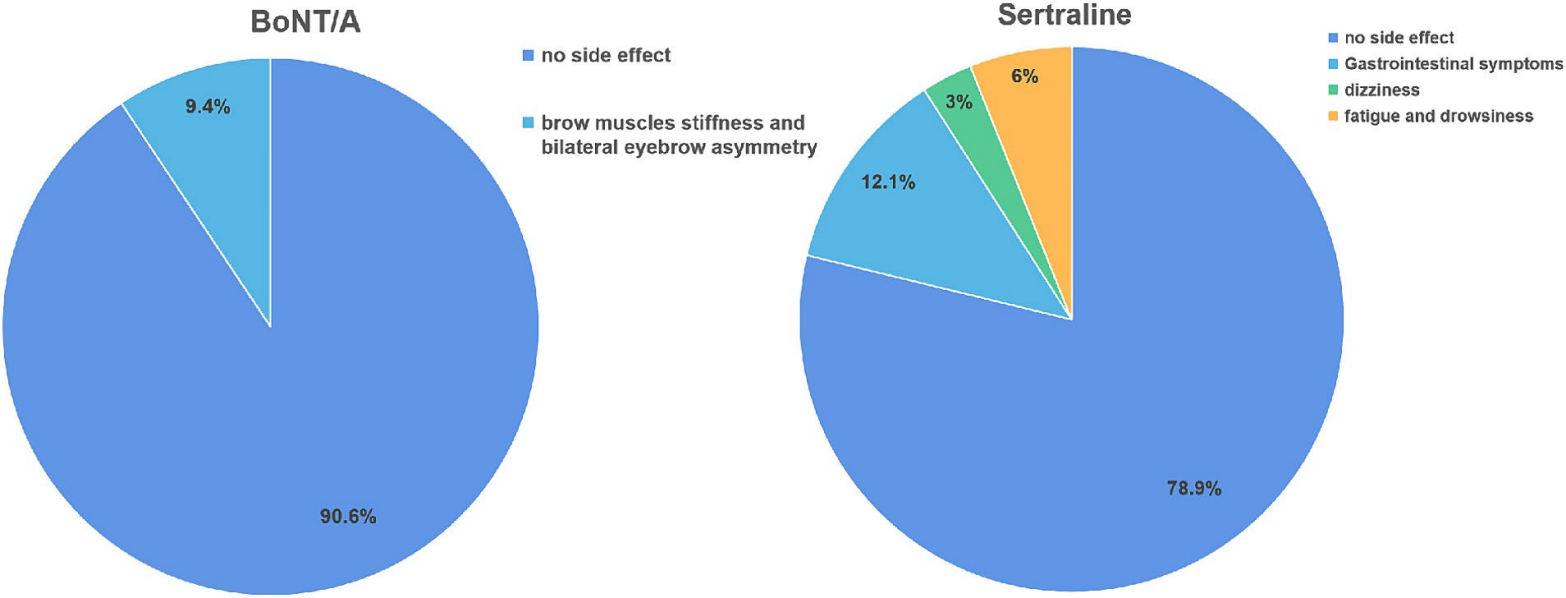
Comparison of side effects between two groups of PSD patients after treatment. BoNT-A, Botulinum Toxin A.

Out of the 32 PSD patients treated with BoNT-A, 3 experienced symptoms of eyebrow stiffness, and 1 had asymmetrical eyebrows. In most cases, these adverse reactions resolved within 3 weeks. Among the 33 PSD patients receiving Sertraline treatment, 4 experienced gastrointestinal reactions, such as dyspepsia and nausea, 1 reported dizziness, and 2 reported fatigue and weakness. Generally, these adverse reactions disappeared within 2 weeks once the patients developed drug tolerance (Figure 4).

## Discussion

The findings of our study demonstrated that BoNT-A effectively reduced depression in PSD patients, as evidenced by significant reductions in psychological scale scores following local injection. The therapeutic effect of BoNT-A on both depression and anxiety was comparable, as indicated by improvements in HAMD and HAMA scores after just 1 week of treatment. Furthermore, BoNT-A treatment exhibited efficacy that was comparable to the traditional antidepressant Sertraline in the positive control group, while also being associated with fewer side effects.

We made careful adjustments and improvements in selecting the injection sites and dosage based on previous research. In the clinical study conducted in 2020 by Brin et al. (12), doses of 30 U and 50 U of BoNT-A were used for local injections to treat depression, but the observed effect was not significant. In our team’s previous research (13, 14), the injection dosage were changed to 100 U and we found BoNT/A demonstrated statistically significant antidepressant superiority over placebo. Therefore in this study, we administered 100 U of BoNT-A at 20 sites in the frontal region, bilateral lateral canthus, and temporal region. The targeted negative expression muscles, such as the corrugator and procerus muscles, are associated with unhappy expressions. In addition to these specific muscles, we included the bilateral lateral canthus and bilateral temporal areas to target paralyzed muscles that may block the afferent function of the trigeminal nerve. This blocking effect may reduces the coupling between the brainstem and the left amygdala. However, further investigation is still needed to explore lower, safer, and more effective doses for treating patients. The pathogenesis of PSD is currently the subject of various theories, encompassing neuroanatomy, neurotransmitters, neuroendocrine functions, inflammatory reactions, and psychological factors. Emerging research indicates that PSD’s pathogenesis may involve the disruption of the monoaminergic system, neuroinflammation, and other molecular mechanisms, including reduced expression of brain-derived neurotrophic factor (BDNF).

Ischemic lesions may interrupt axons containing biogenic amines ascending from the brainstem to the cerebral cortex, resulting in reduced availability of biogenic amines in the marginal structures of the frontal and temporal lobes as well as in the basal ganglia. Depression may be associated with low levels of monoamine substances, particularly 5-HT, NE, and dopamine (15, 16). Recent research has highlighted the role of neuroinflammation in the pathogenesis of PSD. Activation of microglia and astrocytes, triggered by PSD, leads to an increase in inflammatory factors such as TNF-α, IL-1β, and IL-6 (17, 18). In a mouse model of PSD, Di Wu et al. observed a significant rise in IL-8, mediated by the IL-18 receptor/NKCC1 signaling pathway (18). Cytokines have been implicated in the pathophysiology of acute stroke and depression, suggesting their involvement in the development of PSD (19). Moreover, clinical studies have revealed a down-regulation of complement which is a precursor transmitted by cytokines of PSD patients (20). Cytokines can up-regulate the expression of the indoleamine 2,3-dioxygenase (IDO) gene, leading to the synthesis of tryptophan to kynurenine, resulting in a decrease in 5-HT synthesis and an increase in neurotoxic tryptophan metabolites in PSD patients. Consequently, 5-HT could play a role as a pathogenic factor in PSD (21). Additionally, recent clinical research has identified a strong correlation between serum BDNF levels and PSD, with decreased levels observed in PSD patients (22).

Our team’s recent clinical research has shown that BoNT-A has a certain therapeutic effect on patients diagnosed with major depressive disorder (23–25). Despite this, its exact therapeutic mechanism remains unclear. It is hypothesized that the mechanism may be associated with the theory of “facial feedback” and the modulation of neurotransmitters in the brain. According to the facial feedback theory, facial expressions are interconnected with emotional perception. When a sad expression is displayed, the afferent fibers of the peripheral muscles send signals of unhappiness to the brain. In a clinical study, the injection of BoNT-A into the frowning muscle led to improved activity in the left amygdala (26). BoNT-A acts on the motor nerve terminals, blocking the release of the neurotransmitter acetylcholine by interrupting the signal transmission at the neuromuscular junction, resulting in localized muscle paralysis. Consequently, localized administration of BoNT-A can improve one’s overall facial expression, positively influencing emotional perception in the brain.

Our previous experimental investigations have shown that BoNT-A can elevate the levels of 5-HT and BDNF in the brain, thereby mitigating depressive behavior in mice through the activation of the BDNF/ERK/CREB pathway (27). Other experimental studies have similarly demonstrated that local injections of BoNT-A in rats can enhance the metabolism of monoaminergic neurotransmitters in the brain’s limbic system and increase the expression of BDNF. Notably, elevated levels of 5-HT in the hypothalamus and NA in the striatum were observed in the BoNT-A-treated group (BoNT-A + saline) compared to the control group (saline + saline) (28). This finding suggests that the mechanism underlying BoNT-A’s treatment of depression may be associated with its ability to locally increase 5-HT and NA levels in the brain. Furthermore, a study investigating neuropathic pain (NP) revealed that the combination of BoNT-A and MC can enhance the expression of sirt1, which subsequently suppresses the expression of p53, pAKT, and p-NF-KB, thus attenuating oxidative stress and inflammatory response in the glial cell (29). Recent evidence suggests that BoNT-A has significant effects in the treatment of depression. However, there is currently no research on the use of BoNT-A in treating PSD. Our study found that BoNT-A also had a significant effect on patients with PSD, likely due to its ability to block the transmission of cholinergic neurons. As a conventional SSRI antidepressant that we selected Sertraline as the positive control group and compared the treatment efficacy and side effects between the two groups. Upon comparison, we found that BoNT-A exhibited equivalent efficacy to Sertraline in treating PSD, but with less severe side effects. Previous research has reported that the adverse effects of BoNT-A are generally limited to transient and short-lived symptoms such as headache, dry eye, eyelid edema, and periocular muscle spasm, among others. These findings are consistent with our results, indicating that BoNT-A is a safe and reliable treatment option. Moreover, the effects of BoNT-A injections can last for 2–3 months, improving patient compliance and reducing side effects compared to Sertraline.

Following the study, BoNT-A demonstrated statistically the efficacy and safety in treating patients with PSD, thus presenting a novel treatment approach for the clinical management of PSD. There are several limitations of our study remaining to improve in the following research. First, a double-blind study design and placebo control should be implemented to raise the level of research evidence and the reliability of the results. Also, the inclusion of patients with severe PSD, the expansion of the sample size, and further exploration of the underlying mechanisms by which BoNT-A effectively treats PSD need to be considered in the future.

## Data availability statement

The original contributions presented in the study are included in the article/supplementary material, further inquiries can be directed to the corresponding authors.

## Ethics statement

The studies involving humans were approved by the Ethics Committee of the Second Affiliated Hospital of Suzhou University. The studies were conducted in accordance with the local legislation and institutional requirements. The participants provided their written informed consent to participate in this study.

## Author contributions

X-YF: Writing – original draft, Supervision, Project administration, Methodology, Funding acquisition, Conceptualization. T-TS: Writing – original draft, Project administration, Investigation, Methodology, Conceptualization. Q-CW: Writing – original draft, Methodology, Conceptualization. JW: Writing – original draft, Formal analysis. PN: Writing – review & editing, Validation. JL: Writing – review & editing, Validation. X-PZ: Supervision, Writing – review & editing. HH: Writing – review & editing, Supervision. W-FL: Methodology, Funding acquisition, Conceptualization, Writing – review & editing.
